# Supplementary material for: Acanthamoeba keratitis cases in Sweden: genotypes and clinical course
Source: Microbiol Spectr. 2026 Mar 31;14(5):e03058-25. doi: 10.1128/spectrum.03058-25 (PMC13142033; doi:10.1128/spectrum.03058-25)
Supplement: Supplemental material — Tables S1 and S2. [file spectrum.03058-25-s0001.docx]

Table 1 Addendum. List of *Acanthamoeba* reference sequences

| **Sequence type, Accession number, ATCC number** |
| --- |
| T1, U07400 |
| T1, GQ924681 |
| T2, APU07411, ATCC30870 |
| T2, AF019050 |
| T2_6A, AY026244, ATCC30872 |
| T2_6A, AB425949 |
| T2_6B, AB425945 |
| T2_6B, AY173011 |
| T2_6C, AF019051 |
| T2_6C, HM159368 |
| T2_6C, AM408799 |
| T3, U07412, ATCC30731 |
| T3, AF019053 |
| T4, U07410, ATCC50497 |
| T4A, AF019055 |
| T4A, U07413 |
| T4B, U07414, ATCC50370 |
| T4B, AF019057 |
| T4C, U07409, ATCC50369 |
| T4D, AY361644, ATCC30973 |
| T4D, EF429131 |
| T4E, AF019060 |
| T4E, AF019061, ATCC30871 |
| T4F, AF346662, ATCC50254 |
| T4-Neff, U07416, ATCC50373 |
| T5, U94739 |
| T5, U94732 |
| T6, AF019063, ATCC50708 |
| T6, AF251939 |
| T7, AF019064, ATCC30137 |
| T7, DQ992178 |
| T8, AF019065 |
| T9, AF019066, ATCC30135 |
| T10, AF019067 |
| T10, GU808320 |
| T11, AF019068, ATCC30730 |
| T11, AF019069 |
| T12, AF019070, ATCC30866 |
| T12, EU686716 |
| T13, AF132134 |
| T13, AF132136 |
| T14, AF333609 |
| T14, AF333607 |
| T15, KY513793 |
| T15, AY262360, ATCC30732 |
| T16, AY026245 |
| T16, GQ380408 |
| T17, GU808277 |
| T18, KC822461 |
| T19, KJ413084 |
| T19, KP711387 |
| T20, DQ451161 |
| T21, KX840327 |
| T23, MZ272148 |

Table 2 Addendum. Results of sequence analysis with BLAST nucleotide database for Swedish *Acanthamoeba* specimens

| **Specimen number** | **Sequence type** | **Accession number of sequences with significant alignment (100% coverage) and percent identity** | **257 bp region of 18S rDNA** | **1375 bp region of 18S rDNA** |
| --- | --- | --- | --- | --- |
| PHAS6-1 KLAR | T4E | KY934458; 98,68% | X |  |
| PHAS7-1 KLAR | T4A | HM036181; 99,52% | X |  |
| PHAS7-2 KLAR | T3 | AF019052; 99,56% | X | X |
| PHAS8-1 KLAR | T4A | KT735331 and KP677460 and KM099394; 100% | X |  |
| PHAS10-2 KLAR | T4A | MF176166 and KX714007 and KX688031; 99,55% | X |  |
| PHAS13-1 KLAR | T4B | KY072778 and AY026248; 100% | X |  |
| PHAS17-1 KLAR | T4B | KY072778 and KT735329; 100% | X |  |
| PHAS18-1 KLAR | T3 | KY072779 and S81337; 99,75% | X | X |
| PHAS18-2 KLAR | T3 | KF010846; 100% and AGU07412; 99,70% | X | X |
| PHAS18-3 KLAR | T4A | MN153012 and KX688033; 99,55% | X |  |
| PHAS18-4 KLAR | T6 | AY033896; 99,22% and AF251939; 97,37% | X | X |
| PHAS18-5 KLAR | T3 | AF019052; 99,45% | X | X |
| PHAS20-1 KLAR | T4A | JX423610 and JX441873; 100% | X |  |
| PHAS21-1 KLAR | T4A | MG825456 and GU320593; 99,66% | X |  |
| PHAS21-2 KLAR | T4E | KY934458; 99,05% | X | X |
| PHAS22-1 KLAR | T4A | MG825456; 98,75% and AF260720; 98,75% and U07410; 98,75% | X |  |
